# Annual change in eGFR in renal hypouricemia: a retrospective pilot study

**DOI:** 10.1007/s10157-024-02558-8

**Published:** 2024-10-03

**Authors:** Shinobu Sugihara, Yasutaka Yamamoto, Kei Teramoto, Toshiro Hamada, Satoshi Miyazaki, Kazuhide Ogino, Masanari Kuwabara, Akira Ohtahara, Einosuke Mizuta, Kimiyoshi Ichida, Yusuke Endo, Hiroyuki Minato, Haruaki Ninomiya, Masahiko Kato, Kazuhiro Yamamoto, Ichiro Hisatome

**Affiliations:** 1 Matsue Health Service Center, Shimane University, Matsue, Japan; 2 Yasutaka Yamamoto Cardiology/Internal Medicine Clinic, Fuji, Japan; 3 The Division of Medical Informatics, Tottori University Hospital, Tottori, Japan; 4 Department of Community-Based Family Medicine, Faculty of Medicine, Tottori University, Yonago, Japan; 5 Division of Cardiology, Fujii Masao Memorial Hospital, Kurayoshi, Japan; 6 Department of Cardiology, Japanese Red Cross Tottori Hospital, Tottori, Japan; 7 Department of Cardiology, Toranomon Hospital, Tokyo, Japan; 8 Department of Cardiology, Sanin Rosai Hospital, Yonago, Japan; 9 Department of Pathophysiology, Tokyo University of Pharmacy and Life Sciences, Tokyo, Japan; 10 Advanced Medicine, Innovation and Clinical Research Center, Tottori University Hospital, Yonago, Japan; 11 Department of Anesthesiology, Faculty of Medicine, Tottori University, Yonago, Japan; 12 Department of Biological Regulation, Tottori University, Yonago, Japan; 13 Division of School of Health Science, Department of Pathobiological Science and Technology, Faculty of Medicine, Tottori University, Yonago, Japan; 14 Department of Cardiovascular Medicine and Endocrinology and Metabolism, Faculty of Medicine, Tottori University, Yonago, Japan; 15 Department of Cardiology, National Hospital Organization, Yonago Medical Center, Yonago, Japan

**Keywords:** Renal hypouricemia, URAT1 gene mutation, Kidney dysfunction, eGFR

## Abstract

**Background:**

Extremely low uric acid (UA) levels or increased urinary UA (Uua) excretion might be risk factors for kidney disease in renal hypouricemia (RHU) patients, but their relationship with kidney dysfunction is unclear. This study investigated time-dependent changes in eGFR in RHU patients.

**Methods:**

This multicenter retrospective study assessed UA metabolism and changes in eGFR (median 5.5 years) in 13 RHU patients. We then compared eGFR change in 7 of 13 RHU patients whose eGFR could be measured for 4 years with those in normouricemic group (*n* = 31). In addition, 7 RHU patients were divided into two groups based on URAT1 gene mutations: homozygote and compound heterozygote mutations (Homo/Com group, *n* = 3), and wild-type and heterogeneous mutations (WT/Hetero group, *n* = 4).

**Results:**

In 13 RHU patients, the median and mean serum UA (SUA) were 0.8 (0.4–2.5) and 1.1 ± 0.7 mg/dL. The median and mean Uua were 44.3 (12.7–141.1) and 49.7 ± 36.2 mg/dL. The median and mean urinary urate clearance (Cua/Ccr) were 46.8 (11.3–73.6) and 43.3 ± 19.7%. Over 4 years, eGFR did not change in the RHU group but declined in the normouricemic group. Annual mean eGFR decline and change rate in the RHU group were the same as those in the normouricemic group (− 1.09 ± 1.11 vs. − 1.09 ± 1.92 mL/min/1.73 m^2^/year, *p* = 0.996) (− 1.74 ± 1.96 vs. − 1.36 ± 2.10%, *p* = 0.664). And no significant difference was found in eGFR decline or change rate between Homo/Com and WT/Hetero groups (− 0.33 ± 1.03 vs. − 1.67 ± 0.85 mL/min/1.73 m^2^/year, *p* = 0.116) (− 0.61 ± 1.62 vs. − 2.59 ± 1.91%, *p* = 0.210).

**Conclusion:**

RHU from URAT1 genetic mutation may not show eGFR decline over 4 consecutive years.

## Introduction

Hypouricemia is defined as serum uric acid (SUA) level ≤ 2 mg/dL [1]. Several cross-sectional studies have reported an association between low SUA levels and renal dysfunction. Extremely low UA levels or high urinary UA (Uua) excretion may be risk factors for kidney disease in renal hypouricemia (RHU) patients; however, reports on the effects of RHU on kidney function and prognosis remain controversial. Wakasugi et al. [2] reported that hypouricemia is associated with decreased renal function in males. Kuwabara et al. [3] reported that males with hypouricemia were nine times more likely to have a history of kidney disease than those without hypouricemia. However, these studies did not clarify the causal relationship between hypouricemia and kidney function. In a committee-based prospective cohort study, Kanda et al. [4] reported that low SUA levels (male < 5 mg/dL; female < 3.6 mg/dL) were associated with a time-dependent decline in eGFR. A recent prospective cohort study by Ueda et al. [5] showed that a lower SUA level (2.0–2.9 mg/dL) was related to an increased risk for a rapid decline in kidney function (△eGFR ≥ 3 mL/min/1.73 m^2^/year). Mori et al. [6] showed that low SUA (≤ 3.5 mg/dL) was a risk factor for chronic kidney disease (CKD) in females but not in males during a 10-year period. In contrast, Koto et al. [7] found no relationship between hypouricemia and kidney dysfunction. Therefore, the association between low SUA levels and poor kidney function remains controversial, possibly due to the genetic variations in RHU. However, no studies have evaluated the annual changes in the eGFR of patients with RHU proven by genetic analysis.

Most hypouricemia cases involve RHU, affecting about 0.3% of the Japanese population [1]. RHU is a genetic disorder caused by mutations in UA transporters (UATs). Ninety percent of the UA filtered in the glomerulus is reabsorbed by UATs such as *URAT1/SLC22A12* [8], localized on the apical side of the proximal tubules, and *GLUT9/SLC2A9* [9], localized on the basolateral membrane side. Impaired UA reabsorption induces excess urinary excretion, resulting in RHU [10]. In patients with RHU, exercise-induced acute kidney injury (EIAKI) is among a notable complication, occurring in 6.5–24.1% of RHU cases [8, 10, 11]. Most patients with both RHU and EIAKI have URAT1 or GLUT9 gene mutations, showing fractional excretion of UA (FEUA) at > 30% [8]. EIAKI, caused by renal tubule damage from high UA after exercise, is prevented by xanthine oxidase inhibitors (XOIs) [12]. Although EIAKI usually resolves within 2 weeks, approximately 40% of patients experience relapses [10], which may lead to progressive eGFR decline and CKD [13]. The long-term impact of RHU on renal function remains unclear.

We examined UA metabolism and decline in eGFR in RHU patients with URAT1 gene mutation and found that their eGFR decline was similar in normouricemic individuals. This report is placed as a pilot study for evaluating the kidney function prognosis in genetically confirmed RHU patients.

## Methods

### Participants and study design

Hypouricemia is generally defined as SUA at ≤ 2.0 mg/dL. Although patients with an SUA level ≤ 2 mg/dL and an increase in urinary urate clearance (Cua/Ccr) are involved in RHU, the guidelines for managing RHU classify an SUA level ≤ 3 mg/dL associated with increased Cua/Ccr as RHU [1]. Thus, in the present study, RHU was defined as SUA level ≤ 3 mg/dL and increased Cua/Ccr (> 10%). Thirteen RHU outpatients (males: 46.2%) had visited regularly Tottori University Hospital or NHO Yonago Medical Center and had examinations at least once a year with a median follow-up of 5.5 (3.0–7.3) years. Among the 31 normouricemic individuals (3.0 < SUA < 7 mg/dL) visiting NHO Yonago Medical Center for health check-up (males: 58.1%), those whose both age and sex were matched with those in the RHU group were retrospectively enrolled as individuals in the normouricemic group. First, we examined the UA metabolism, complications, and changes in eGFR (median 5.5 years) in 13 RHU patients. Baseline data, including age, sex, body mass index (BMI), complications such as hypertension (HT), diabetes, dyslipidemia (DL), urolithiasis, EIAKI, laboratory data, and medications, were obtained from medical records. Venous blood and urine samples were collected after an overnight fast. Blood and urine biochemical data were analyzed using an autoanalyzer. SUA, Uua, serum creatinine (Scr), and urinary creatinine (Ucr) levels were measured using the uricase-POD method to evaluate UA metabolism. The Cua/Ccr ratio was determined as previously described [14]. Uua/Ucr is an index of UA production, and Cua/Ccr is an index of UA excretion without reabsorption in the proximal tubules. Genetic analysis of URAT1 was performed as previously described by Sugihara et al. [15] was reused in patients with RHU, and eGFR for the Japanese population was calculated using the following equation: eGFR (mL/min/1.73 m^2^) = 194 × serum Cr-1.094 × age-0.287 (for females) × 0.739, where Cr = serum creatinine level (mg/dL) [16].

Second, since eGFR was measured over 4 consecutive years in 7 of the 13 RHU patients, we compared the characteristics and changes in eGFR over 4 consecutive years between the patients with RHU and 31 normouricemic individuals. Subsequently, we classified patients with RHU into two groups based on URAT1 gene mutations: homozygote and compound heterozygote mutations (Homo/Com group), and wild-type and heterogeneous mutations (WT/Hetero group). The eGFR decline per year was calculated using the following formula: (eGFR_5th_ − eGFR_1st_)/4, and the rate of change in GFR over 4 years per year was calculated as (eGFR_5th_ − eGFR_1st_)/eGFR_1st_/4 × 100.

The Institutional Review Boards of Tottori University Hospital and Yonago Medical Center approved the study protocol (approval numbers: 21A048 and 040611, respectively). Informed consent was obtained in the form of opt-out. Patient privacy is protected in accordance with the Declaration of Helsinki.

### Statistical analysis

Numeric data were expressed as the mean ± standard deviation (SD) in normally distributed data and median (minimum and maximum value) in non-normally distributed data. Categorical data were expressed as numbers and percentages. Differences in the continuous variables between the two groups were compared using parametric analysis. Changes between the groups over time were evaluated using repeated-measures analysis of variance, Bonferroni’s multiple comparisons of repeated-measures analysis of variance, and the Greenhouse–Geisser method. *p* < 0.05 was considered statistically significant. Statistical analyses were performed using EZR version 1.64 (Saitama Medical Center, Jichi Medical University, Japan) [17].

## Results

### Demographic data and change in eGFR in 13 patients with RHU

**Fig. 1:**
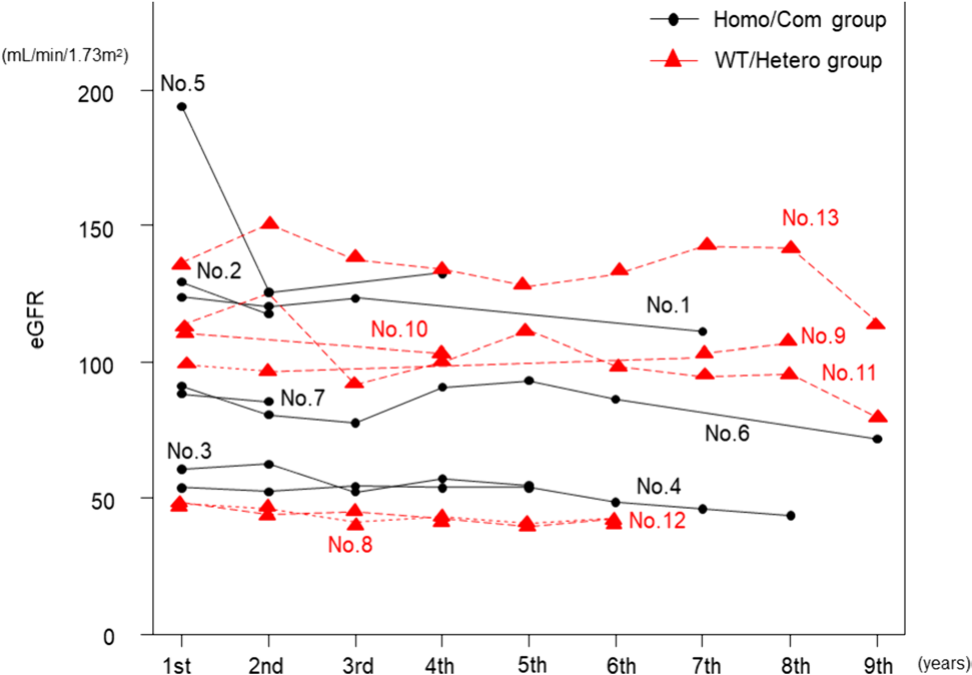
Changes in eGFR of 13 patients with RHU over time. The median follow-up was 5.5 years, with gradual changes over time. Number in each graph is case number in Table 1. Homo/Com group: homozygote and compound heterozygote mutant of URAT1 gene. WT/Herero group: wild-type and heterozygous mutant of URAT1 gene

Table 1 shows demographic data on UA metabolism in 13 patients with RHU, indicating that the median and mean SUA values were 0.8 (0.4–2.5) and 1.1 ± 0.7 mg/dL, respectively. The median and mean Uua, Uua/Ucr, and Cua/Ccr values were 44.3 (12.7–141.1) and 49.7 ± 36.2 mg/dL, 0.50 (0.42–0.86) and 0.57 ± 0.16, and 46.8 (11.3–73.6) and 43.3 ± 19.7%, respectively. For URAT1 mutations, seven patients had a null mutation (Homo/Com group), two harbored a heterogeneous gene mutation, and three harbored the wild-type gene. Genetic analysis was not performed in one patient (WT/hetero group). Three patients in the homo/Com group and one patient in the WT/hetero group had urolithiasis. Overall, two patients had HT, and one each had Sjögren’s syndrome, EIAKI, and DL. Figure 1 shows the changes in the eGFR of the 13 patients with RHU over time, showing a gradual variation in eGFR.

**Table 1:** Demographic data on uric acid metabolism in 13 patients with RHU

No	Age	Sex	URAT1 mutation	Group	Complication		BMI (kg/m^2^)	SUA (mg/dL)	Scr (mg/dL)	eGFR (mL/min/1.73 m^2^)	Uua (mg/dL)	Ucr (mg/dL)	Uua/Ucr	Cua/Ccr (%)
					Urolithiasis	Others								
1	63	Male	G774A homo	Homo/Com	+	Sjögren’s syndrome	23.2	0.4	0.59	105.2	29.6	59.6	0.50	73.6
2	38	Male	G774A homo	Homo/Com	–	EIAKI	29.7	0.7	0.57	146.1	79.7	117.6	0.68	55.2
3	75	Male	G774A homo	Homo/Com	+	HT	27.7	0.8	1.00	53.8	24.7	49.1	0.50	62.9
4	66	Male	G774A/G269A compound hetero	Homo/Com	–	–	23.6	0.9	1.10	54.2	66.7	155.7	0.43	52.4
5	34	Female	G774A /IVS2 + 1G compound hetero	Homo/Com	–	–	19.7	0.5	0.31	202.7	83.5	99.4	0.84	52.1
6	71	Male	626 T/1137G compound hetero	Homo/Com	–	–	20.8	0.7	0.73	82.6	50.9	120.6	0.42	44.0
7	33	Female	V138M/W258X compound hetero	Homo/Com	+	–	21.6	0.6	0.58	95.0	52	107.3	0.48	46.8
8	70	Female	URAT1 rs2039388764 (P379L) + GLUT9 rs2276961 (G25R)	WT/Hetero	+	HT, DL	19.6	0.7	1.00	42.5	44.3	104.6	0.42	60.5
9	76	Female	T1289C hetero	WT/Hetero	–	–	16.9	1.4	0.44	101.6	16.8	19.6	0.86	26.9
10	44	Female	Not detect	WT/Hetero	–	–	21.1	2.5	0.48	109.5	12.7	16.2	0.78	15.1
11	75	Female	Wild type	WT/Hetero	–	–	17.4	1.8	0.47	96.7	141.1	326.8	0.43	11.3
12	80	Female	Wild type	WT/Hetero	–	–	16.4	2.5	0.78	54.0	24.9	44.1	0.56	17.6
13	50	Male	Wild type	WT/Hetero	–	–	29.3	0.8	0.63	104.6	19.4	34.3	0.57	44.5

*Homo/Com* homozygous and compound heterozygous mutation groups, *WT/Hetero* wild-type and heterogeneous gene mutations, *EIAKI* exercise-induced acute kidney injury, *HT* hypertension, *DL* dyslipidemia, *BMI* body mass index, *SUA* serum uric acid, *Scr* serum creatinine, *Uua* urinary uric acid, *Ucr* urinary creatinine, *Cua/Ccr* ratio of urate clearance to creatinine clearance

### Demographic data and change in eGFR between RHU and normouricemic groups

Table 2 shows the baseline characteristics of the seven patients with RHU (case numbers: 3, 4, 6, 8, 11, 12, and 13) compared with those of the 31 normouricemic individuals. Patients with RHU had lower SUA levels, lower prevalence of dyslipidemia, and higher prevalence of urolithiasis than those in the normouricemic group.

**Table 2:** Characteristics of RHU and normouricemic groups

	RHU group *n* = 7 (No 3, 4, 6, 8, 11, 12, 13)	Normouricemic group *n* = 31	*p*
Age (years)	70 ± 10	67 ± 5	0.250
Male (%)	57.1	58.1	0.966
BMI (kg/m^2^)	22.1 ± 5.0	23.4 ± 3.8	0.443
SUA (mg/dL)	1.17 ± 0.70	5.32 ± 1.02	< 0.001
Scr (mg/dL)	0.82 ± 0.23	0.76 ± 0.18	0.452
eGFR (mL/min)	69.8 ± 24.5	73.2 ± 13.5	0.614
TG (mg/dL)	123.3 ± 64.0	89.9 ± 34.0	0.057
HDL-C (mg/dL)	68.0 ± 23.0	70.5 ± 17.0	0.746
LDL-C (mg/dL)	106.6 ± 29.2	118.2 ± 24.0	0.273
AST (IU/L)	24.0 ± 5.8	21.6 ± 4.6	0.239
ALT (IU/L)	19.0 ± 7.6	19.4 ± 6.3	0.888
γ-GTP (IU/L)	43.9 ± 44.7	34.0 ± 22.4	0.395
Dyslipidemia (%)	14.3	61.3	< 0.05
Hypertension (%)	28.6	48.4	0.354
Diabetes (%)	0.0	19.4	0.215
Urolithiasis (%)	28.6	0.0	< 0.05

The RHU group consisted of six patients (Nos. 3, 4, 6, 8, 11, 12, and 13)

*BMI* body mass index, *SUA* serum uric acid, *Scr* serum creatinine, *TG* triglyceride, *HDL-C* high-density lipoprotein cholesterol, *LDL-C* low-density lipoprotein cholesterol

**Fig. 2:**
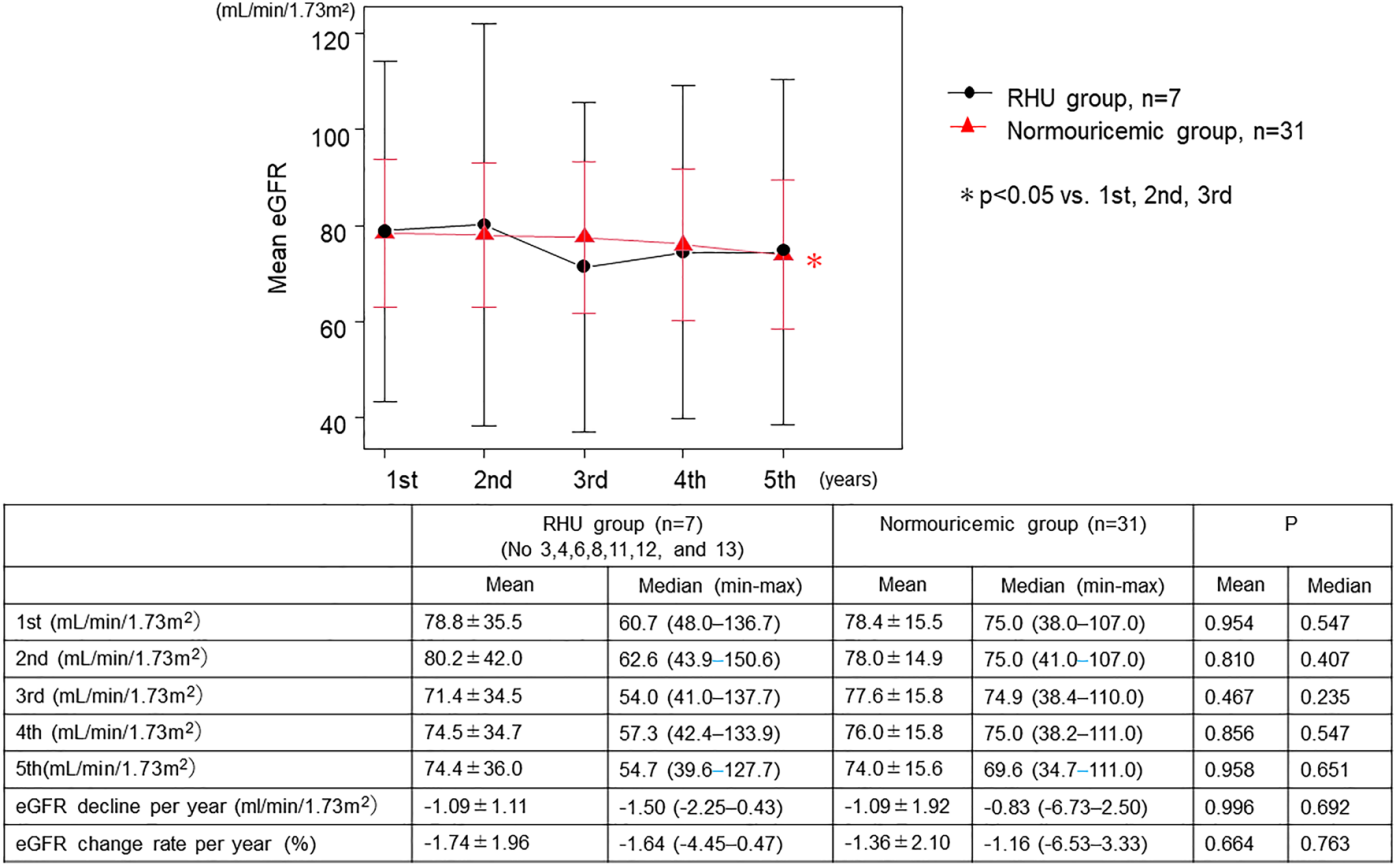
Changes in eGFR between the RHU and normouricemic groups for 4 consecutive years. The upper graph shows the change in mean eGFR over time, and the lower table shows the mean and median (minimum–maximum) eGFR values for each year, the difference in eGFR decline per year, and the eGFR change rate per year in both groups. * *p* < 0.05 vs. 1st, 2nd, and 3rd year

Figure 2 shows the differences in eGFR between the two groups. In normouricemic individuals, eGFR at 5th year was predominantly lower than at 1st, 2nd, and 3rd years, whereas in patients with RHU, eGFR did not change until the 5th year. No differences were observed in the mean and median eGFR between the two groups each year. In addition, the mean and median eGFR decline per year and eGFR change rate per year showed no significant differences between the two groups.

### Change in eGFR in *Homo*/Com and WT/Hetero groups of patients with RHU

**Fig. 3:**
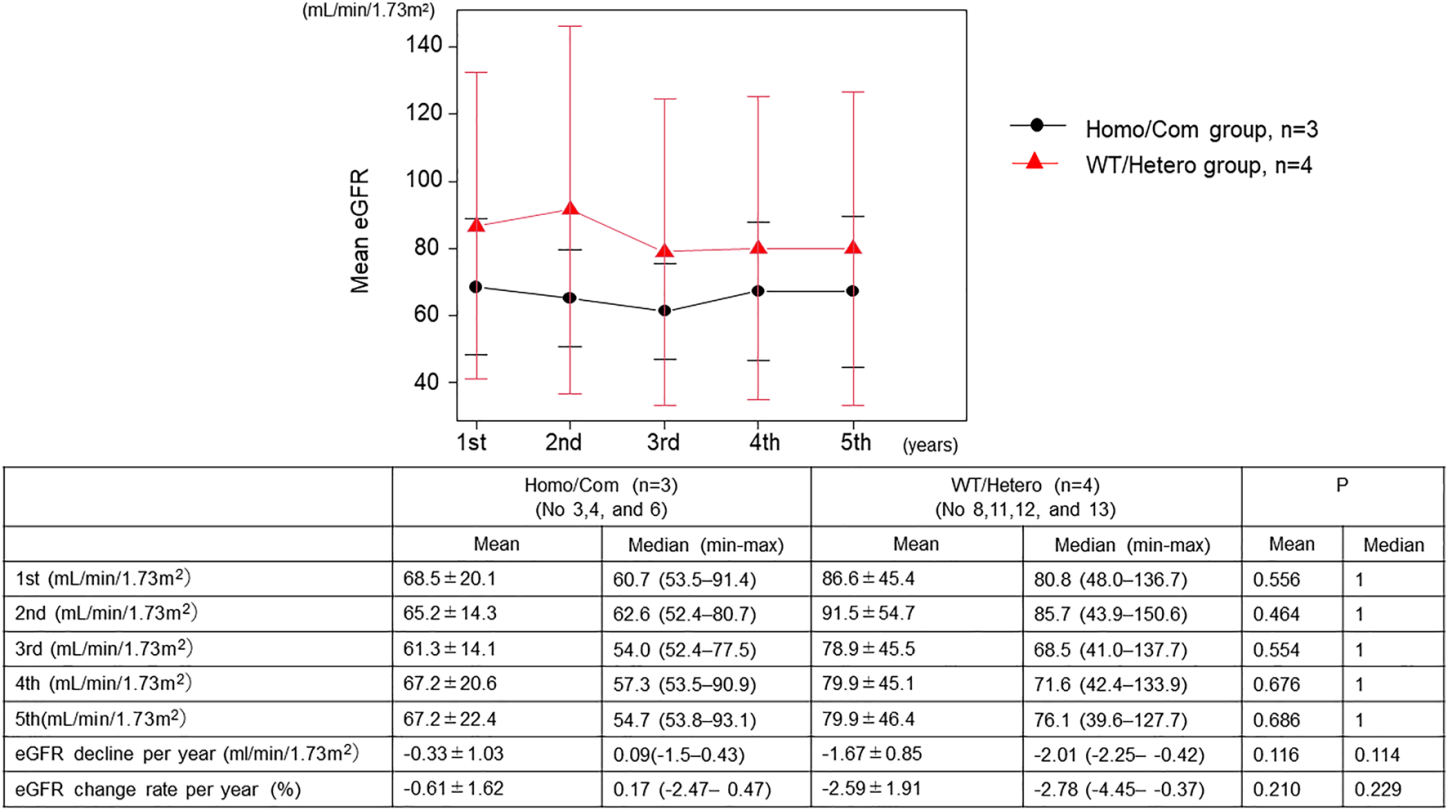
Changes in eGFR between Hono/Com group and WT/Hetero group for 4 consecutive years. The upper graph shows the change in mean eGFR over time and the lower table shows the mean and median (minimum–maximum) eGFR values for each year, the difference in eGFR decline per year, and the eGFR change rate per year in both groups

Of the seven patients with RHU, three belonged to the Homo/Com group (case numbers: 3, 4, and 6), and the other four belonged to the WT/Hetero group (case numbers: 8, 11, 12, and 13). As shown in Fig. 3, no changes in the mean and median eGFR over time were observed between the two groups, and no significant difference was observed in the eGFR each year between the two groups. No significant differences were observed in the mean and median eGFR decline per year and the rate of change per year between the two groups.

## Discussion

In the present study, we found that (1) eGFR in patients with RHU was comparable to that in normouricemic subjects during the observation period, and (2) no difference was observed in eGFR decline between the Homo/Com and WT/Herero groups in patients with RHU.

RHU due to the impaired UATs (URAT1 [8], GLUT9 [9]) is characterized by SUA at ≤ 3 mg/dL and increased Cua/Ccr (>10%). RHU is associated with complications, such as urolithiasis [18], EIAKI [11, 12], and kidney dysfunction [6]. Four possible mechanisms have been reported for the hypouricemia-induced impairment of kidney function. First, oxidative stress in patients with RHU may induce arterial vasoconstriction in the kidney by impairing nitric oxide (NO)-dependent and endothelium-derived hyperpolarizing factor (EDHF)-mediated vasodilation [19]. Because hypouricemia attenuates antioxidant action and increases oxidative stress, it causes the degradation of NO and might reduce its ability to promote epoxyeicosatrienoic acid production, which is responsible for EDHF-mediated vasodilation [19]. Second, the loss of blood hypoxanthine after exercise may deplete adenosine triphosphate (ATP) in the renal tubules and cause kidney damage. Patients with RHU have significantly lower blood hypoxanthine levels, which are associated with reduced blood ATP levels after exercise [20]. Third, high concentrations of Uua can induce inflammasome activation in tubules. An experimental study reported that a high concentration of UA in the proximal tubules and thick ascending limb of Henle’s loop stimulates the luminal Toll-like receptor 4 pathway and activates the nucleotide-binding oligomerization domain-like receptor family pyrin domain-containing (NLRP3) inflammasome [12]. The fourth is UA crystal in renal tubules. Elevated Uua levels in patients with RHU may cause intratubular and/or intraurethral precipitation of UA crystals [21], which activate the NLRP3 inflammasome [12, 22].

In the present study, among the 13 patients with RHU, 7 harbored the null mutation of URAT1/SLC22A12, both homozygous and compound heterozygous mutants, and 5 harbored the heterozygous URAT1/SLC22A12 mutation and wild-type URAT1/SLC22A12 gene, one was not performed by genetic analysis. Urolithiasis was associated with four patients (30.8%), and EIAK was associated with one patient (7.7%). Interestingly, these patients harbored a null mutation in URAT1, except for one patient with a mutation in URAT1 and GLUT9 (case number 8, WT/Hetero group). Their Cua/Ccr increased to 44.0 and 73.6%, suggesting that the excess Uua excretion could form UA precipitates and cause urolithiasis, activating tubular NLRP3 inflammasome, thereby causing EIAKI.

The most prominent finding in the present study was that during the observational period for 4 consecutive year, RHU might not have influenced changes in eGFR. Null mutations in URAT1 may not impair changes in eGFR. This is the first study to examine changes in eGFR between patients with and without RHU, and to estimate the impact of a null mutation harboring URAT1 on the decline in eGFR over 4 consecutive years. Prospective cohort study has shown that subjects with SUA levels in the range of 2.0–2.9 mg/dL had a rapid time-dependent decline in eGFR, which was > 3 mL/min/1.73 m^2^/year [5]. The decline in eGFR in healthy individuals was approximately 1% per year, or 0.36–1.25 mL/min/1.73 m^2^/year [23]. In the present study, the mean eGFR decline per year and eGFR change rate per year in 7 patients with RHU and normouricemic individuals were − 1.09 ± 1.11 and − 1.09 ± 1.92 mL/min/1.73 m^2^/year (*p* = 0.996), − 1.74 ± 1.96 and − 1.36 ± 2.10% (*p* = 0.664), respectively. These values were similar to those of healthy subjects. Tabara et al. [24] reported that patients with a nonsense mutation in URAT1 have lower UA levels and impaired renal function. In the present study, however, no difference was observed in mean eGFR decline per year and eGFR change rate per year between the homo/compound heterozygote with URAT1 gene mutation group and wild-type/heterozygote group, − 0.33 ± 1.03 and − 1.67 ± 0.85 mL/min/1.73 m^2^/year (*p* = 0.114), − 0.61 ± 1.62 and − 2.59 ± 1.91% (*p* = 0.210).

These results are inconsistent with those of previous studies. One factor may be the Uua levels. In this study, the Uua levels in patients with RHU ranged from 12.7 to 141.1 mg/dL, and in only one patient, the levels of Uua exceeded 90 mg/dL, the solubility limit of UA at urine pH 6.0 [25], suggesting that the present patients with RHU may not have UA precipitation in the kidney. Besides, recent reports with acute and severe hypouricemia (median SUA was 0.2 mL/dL) indicate that UA reduction may improve endothelial function, blood pressure, and arterial stiffness and enhance antioxidant activity due to decreased myeloperoxidase activity, suggesting that hypouricemia may not always lead to a kidney disease based on impaired endothelial function [26, 27]. However, as these reports dealt with drug-induced hypouricemia, the influence of drugs cannot be excluded; therefore, further investigation on the effects and mechanisms of how RHU impacts long-term renal function is necessary.

Asahina et al. [28] reported that reduced FEUA and increased UA reabsorption in the proximal tubules were linked to CKD progression, suggesting intracellular UA accumulation may damage proximal tubular cells and impair renal function. The present study showed that the annual decline in eGFR in RHU patients with mutant URAT1 gene was comparable to the eGFR levels in normouricemic individuals, indicating potential protective effects of genetic UA reabsorption inhibition, which may support the results of Asahina et al. In the present study, we did not measure Uua and Cua/Ccr levels in normouricemic individuals and could not compare the tubular reabsorption of UA between RHU and normouricemic groups. Further studies are required to clarify this point.

Kawamoto et al. [29] showed that lower total bilirubin levels in patients with high SUA might be a potential risk factor for renal function. As a powerful antioxidant, total bilirubin may influence CKD progression [30]. Although the relationship between total bilirubin and UA has not yet been examined in patients with RHU. In the present study, total bilirubin in the 13 patients with RHU was within the normal limit (0.73 ± 0.26 mg/dL) and the correlation coefficient with SUA was − 0.198 (95% confidence interval (CI): − 0.198 to 0.397; *p* = 0.518) and eGFR was − 0.455 (95% CI: − 0.804 to 0.129; *p* = 0.119). Thus, the role of total bilirubin in renal decline for patients with RHU remains unclear, necessitating further studies.

This study has some limitations. First, owing to the low incidence of RHU, only seven patients with RHU could be compared with normouricemic individuals. Therefore, only univariate comparisons were performed, and multivariate analyses considering age and sex were not possible. Second, sex differences in UA metabolism have been reported [3], but the effects of sex were not analyzed. Third, the observation period of eGFR between RHU and normouricemic groups was short, lasting 4 years. Fourth, muscle mass was not measured in the 13 patients with RHU, which could affect Scr levels and eGFR. However, BMI data in Table 1 showed no significant correlation with eGFR, the correlation coefficient was 0.104 (95% CI: − 0.474 to 0.620; *p* = 0.735) in 13 patients with RHU. In both 7 patients with RHU and 31 normouricemic individuals with eGFR measured for 4 years, no significant correlation between BMI and decline in eGFR was found with the correlation coefficient − 0.095 (95% CI: − 0.381 to 0.208: *p* = 0.540). Collectively, elevated eGFR in RHU patients may not be attributable to low muscle mass, despite some patients having high eGFR values. Finally, because changes in blood pressure, oxidative stress, vascular function, and urine PH other than eGFR were not evaluated, the underlying mechanisms could not be elucidated. The sample size and retrospective study design may restrict its broad applicability to RHU. However, this study is valuable in showing changes in eGFR over time at low UA levels due to RHU.

The novelty of the present study is that eGFR decline in RHU patients was comparable to that in normouricemic individuals, despite reports of higher EIAKI prevalence in RHU. Further research is needed.

## Conclusion

RHU due to URAT1 genetic mutations may not contribute to a decline in eGFR for 4 consecutive years.

## Data Availability

The data used in this study are available from the corresponding author upon request.
